# Change of ankle pain after total knee replacement arthroplasty

**DOI:** 10.1186/1757-1146-7-S1-A134

**Published:** 2014-04-08

**Authors:** Heui-Chul Gwak

**Affiliations:** 1 Department of Orthopedic Surgery, Busan Paik Hospital, College of Medicine, Inje University, 633-165 Gaegeum-dong, Busan Jin-gu, Busan 614-735, South Korea

## Background

We aimed to analyze the change of ankle pain by realignment of the lower extremity after total knee arthroplasty.

## Methods

We performed prospective analysis and followed up 76 patients enrolled from January 2012 to December. 2013 for at least 6 months excepting the 5 patients who were lost follow-up, 71 patients (bilateral: 9, unilateral: 62, total 80 cases) were analyzed. There were 8 men, 63 women and the average age were 69.6 years old (56-79). All surgery were performed by one operator and posterior cruciate ligament stabilized knee arthroplasty was done in all cases. Subjective ankle pain was evaluated by visual analogue scale (VAS) and clinical results were evaluated by AO-FAS ankle-hindfoot scale and SF-36. We described radiologic parameters around the hindfoot and ankle joint including talar tilt, tibial-ankle surface angle, medial clear space, frontal tibial ground angle, frontal talar gound angle, hindfoot alignment view angle and measured the amount of change of knee alignment by comparing preoperative and postoperative X-rays. We divided cases into 4 groups, two with increased ankle pain postoperatively in previous ankle pain group and newly developed pain postoperatively. Pain was aggrevated compared preoperatively was other subgroup, and the other had no change. We compared 4 groups in each parameter and analyzed statistically (SPSS v13.0).

## Results

There was change of ankle pain in 15 cases of 71 patients (80 cases) with over 6 months of follow-up period. Twelve patients had ankle pain before surgery. In 3 cases of them, ankle pain was decreased post-operatively, however, pain of the others was increased postoperatively. Eight cases had newly developed ankle pain postoperatively. In 60 cases, ankle pain had no change. The effect of clinical ankle pain by amount of varus angle correction by total knee arthroplasty showed significant difference between 4 subgroups. There was no significant difference in each parameter between 4 subgroups.

## Conclusion

Change of alignment of lower extremity after total knee arthroplasty can affect on ankle pain.

